# Case Report: Early detection of neonatal volvulus by ultrasound in a 2-day-old neonate: timely intervention prevents complications

**DOI:** 10.3389/fped.2025.1612968

**Published:** 2025-07-18

**Authors:** Yunqin Chen, Hongping Lu, LinJun Yu, Lizhen Wang, Jie Li, Fuen Liang, Haiting Li, Xiyang Chen, Junhui Yuan, Enfu Tao

**Affiliations:** ^1^ Department of Neonatology and NICU, Wenling Maternal and Child Health Care Hospital, Wenling, Zhejiang, China; ^2^ Department of Neonatology, Taizhou Hospital of Zhejiang Province, Wenzhou Medical College, Linhai, Zhejiang, China; ^3^ Department of Pediatric Surgery, Taizhou Hospital of Zhejiang Province, Wenzhou Medical College, Linhai, Zhejiang, China; ^4^ Department of Pediatrics, Taizhou Hospital of Zhejiang Province, Wenzhou Medical College, Linhai, Zhejiang, China; ^5^ Department of Ultrasound, Wenling Maternal and Child Health Care Hospital, Wenling, Zhejiang, China

**Keywords:** intestinal malrotation, volvulus, hematochezia, Ladd's procedure, multidisciplinary collaboration, neonate

## Abstract

Intestinal malrotation is a congenital anomaly arising from improper rotation or fixation of the embryonic gut, potentially leading to life-threatening complications such as volvulus. It typically presents within the first month of life with symptoms including bilious vomiting and scaphoid abdomen. In this report, we describe a case involving a 2-day-old term neonate who exhibited two episodes of hematochezia and one episode of hematemesis, without accompanying scaphoid abdomen or bilious vomiting. Initial laboratory investigations revealed metabolic acidosis (lactate 4.6 mmol/L, base excess −7.28) and positive occult blood (+++). A bedside abdominal ultrasound identified a whirlpool sign, prompting immediate transfer to a tertiary care facility. An emergency laparotomy confirmed a 480 degrees clockwise volvulus without necrosis. The patient underwent a Ladd's procedure and appendectomy, resulting in full recovery. This case represents the earliest documented instance of malrotation presenting with hematochezia and hematemesis within the first 48 hours of life. The absence of necrosis despite gastrointestinal bleeding suggests that hemorrhage in cases of volvulus may precede irreversible ischemia, thereby underscoring the necessity for urgent ultrasound evaluation. We propose that hematochezia in neonates should prompt urgent ultrasound evaluation for malrotation, even in the absence of classic symptoms.

## Introduction

Intestinal malrotation is a congenital anomaly characterized by abnormal positioning of the intestines, resulting from non-rotation, incomplete rotation, or improper fixation of the embryonic gut (1, 2). This condition can lead to a spectrum of clinical presentations, ranging from asymptomatic cases to life-threatening complications such as volvulus, which necessitates prompt surgical intervention (3). The incidence of intestinal malrotation is estimated to be 1 in 500 live births (4), with associated volvulus in 1 in 2,500 live births (2), and midgut volvulus in 42.1% of symptomatic neonatal malrotation cases (5).

Neonates with intestinal malrotation typically present within the first month of life (2), often with bilious vomiting (1, 6, 7), scaphoid abdomen (1, 8) being the most common presentations. Bowel obstruction signs (2), and less frequently, hematochezia or hematemesis (1.79%–8.8%) may also occur (1, 9). This clinical heterogeneity often complicates diagnosis (10), underscoring the imperative for timely intervention to prevent bowel necrosis and associated morbidity (3).

This report describes a 2-day-old term neonate presenting with two episodes of hematochezia and one episode of hematemesis, notably lacking the classic symptoms of scaphoid abdomen or bilious vomiting. Bedside abdominal ultrasound revealed the pathognomonic whirlpool sign, leading to the diagnosis of intestinal malrotation. The patient underwent an emergency Ladd's procedure with appendectomy and achieved full recovery. To our knowledge, this represents the first documented case of intestinal malrotation manifesting as hematochezia and hematemesis within the first 48 hours of life without evidence of bowel necrosis.

## Case description

A 2-day-old female neonate was admitted for two episodes of bloody stools and one of hematemesis. Born at 40 6/7 weeks via uncomplicated vaginal delivery, she weighed 3,300 g, with Apgar scores of 10 at 1 and 5 minutes. The amniotic fluid was clear, and the umbilical cord and placenta were normal. Exclusively breastfed, she had no fever, seizures, or abdominal issues but showed poor feeding. On admission, her temperature was 37.6°C, heart rate 124 bpm, respiratory rate 44 bpm, and blood pressure 69/39 mmHg. The patient was alert, cried strongly, had a flat fontanelle, no rashes, and moderate jaundice. Lung sounds were clear, heart rhythm regular without murmurs, and the abdomen was flat with the liver palpable 1.0 cm below the costal margin; the spleen was not palpable. Primitive reflexes were present.

**Figure 1:**
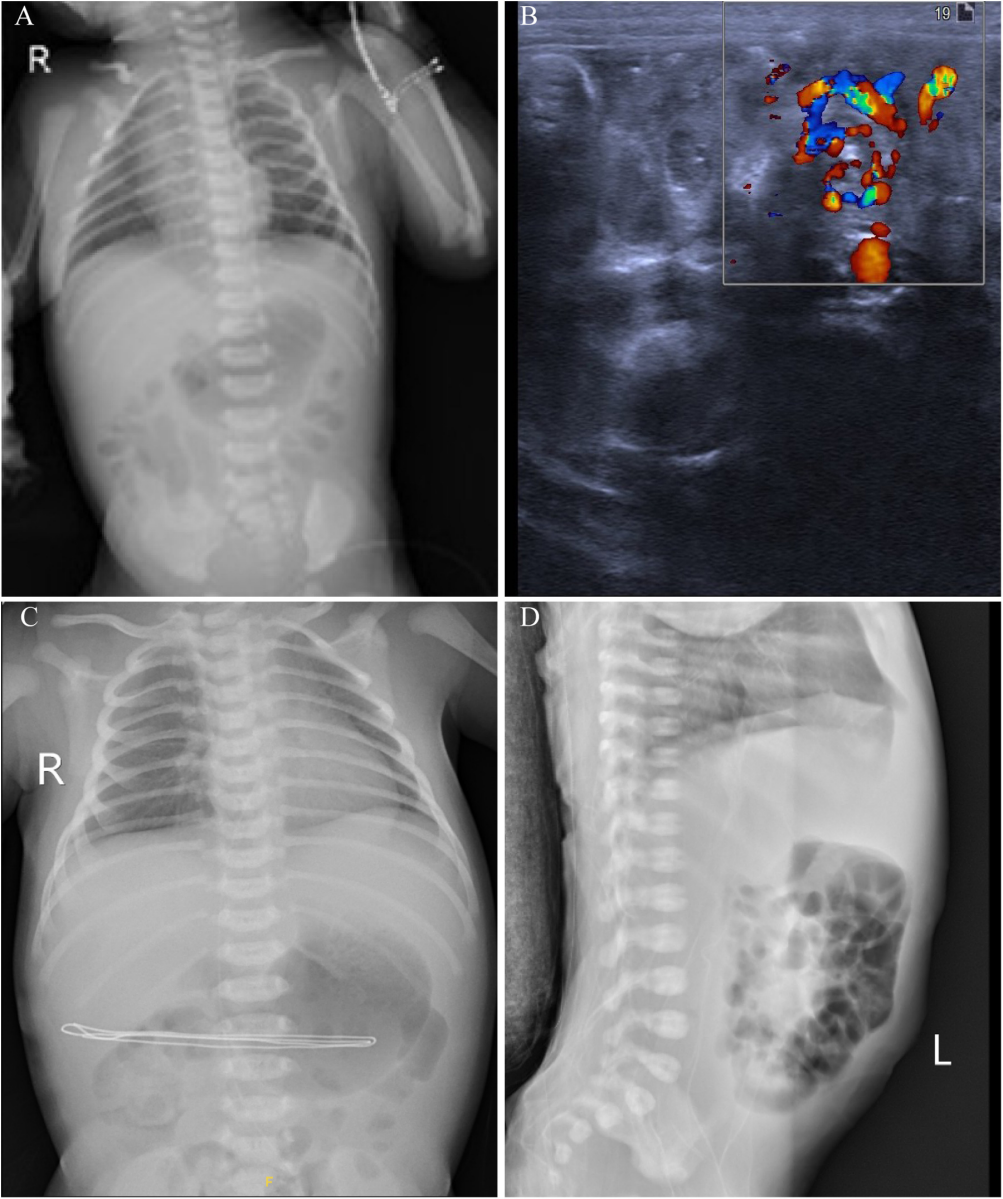
Imaging findings of a 2-day-old neonate with intestinal malrotation. **(A)** Chest and abdominal x-rays show scattered gas in the small intestine and colon, indicating possible gastrointestinal obstruction. **(B)** Abdominal ultrasound reveals the whirlpool sign, with the superior mesenteric vein rotating around the superior mesenteric artery. **(C)** Postoperative x-rays show increased gas in the small intestine and colon on the second postoperative day compared to preoperative levels. **(D)** A lateral abdominal radiograph on the fourth postoperative day shows significant intraluminal gas.

Diagnostic tests revealed a blood gas analysis showing a pH of 7.41, sodium 139.4 mmol/L, calcium 1.17 mmol/L, pCO2 24.2 mmHg, pO2 52.5 mmHg, bicarbonate 14.9 mmol/L, base excess −7.28 mmol/L, blood glucose 5.5 mmol/L, lactate 4.6 mmol/L, and hemoglobin 17.7 g/dl, indicating elevated lactate and negative base excess. The patient received a 30 ml normal saline infusion for volume expansion. Complete blood count (CBC), C-reactive protein (CRP) and coagulation function tests showed no significant issues. Upon admission, the neonate passed a large amount of bright red bloody stool and vomited coffee-colored material. The stool occult blood test was positive (+++). An abdominal x-ray showed gas in the small intestine and colon (Figure 1A), while an ultrasound revealed a mixed mass in the upper abdomen with a “whirlpool sign,” measuring about 21 mm × 18 mm (Figure 1B). Intestinal malrotation was suspected, and the patient was transferred to a tertiary hospital. After ruling out contraindications, the patient underwent an open Ladd's procedure with appendectomy. The surgery was uneventful, revealing approximately 10 ml of brownish fluid in the abdominal cavity, which was sent for culture. The mesenteric root was twisted 480 degrees clockwise, causing midgut volvulus; the small intestine was slightly dark but normalized after untwisting. Immediate fluid resuscitation with crystalloids was initiated intraoperatively to address third-space losses and potential reperfusion injury following untwisting. Hemodynamic parameters were closely monitored, and the patient remained stable throughout the procedure. The surgery was successful, and the patient received antibiotics and nutritional support postoperatively. By the second postoperative day, bedside x-rays showed increased gas in the small intestine and colon compared to preoperative levels (Figure 1C). Subsequently, on the fourth postoperative day, a lateral abdominal radiograph revealed significant intraluminal gas (Figure 1D).

The patient was diagnosed with congenital intestinal malrotation, acute simple appendicitis, neonatal hyperbilirubinemia, and patent foramen ovale. After a 16-day hospitalization, the neonate was discharged in good condition. During one year of follow-up, the patient exhibited excellent growth and development without recurrence. Key laboratory findings and investigations are summarized in Table 1.

**Table 1:** Summary of laboratory investigations and special diagnostic procedures.

Investigation	Results	Reference range	Notes
Complete blood count	11.7	15–20	No evidence of infection
White blood cell, ×10^9^/L			
Neutrophil percentage, %	59.5	20–40	
Lymphocytes percentage, %	26.4	50–75	
C-reactive protein, mg/L	<0.5	0–5.0	
Blood gas			Suggests inadequate tissue perfusion
pH	7.41	7.35–7.45	
Actual bicarbonate, mmol/L	14.9	18.0–26.0	
Base excess, mmol/L	−7.28	−3.0–+3.0	
Lactic acid, mmol/L	4.6	0.5–2.2	
Sodium ion, mmol/L	139.4	135–145	
Potassium ion, mmol/L	4.22	3.5–5.5	
Calcium ion, mmol/L	1.17	1.05–1.35	
Chloride ion, mmol/L	110.5	98–113	
Blood glucose, mmol/L	5.5	3.9–6.1	
Blood biochemistry			Indicates hyperbilirubinemia
Aspartate aminotransferase, IU/L	11	0–50	
Alanine aminotransferase, IU/L	37	0–50	
γ-Glutamyl transferase, IU/L	37	0–50	
Total bilirubin, μmol/L	215.6	0–16.7	
Indirect bilirubin	204.1	0–21.0	
Direct bilirubin	11.5	0–5.0	
Coagulation function	Normal	Normal	Excludes coagulopathy or disseminated intravascular coagulation
Fecal occult blood test	+++	Negative	Suggests gastrointestinal bleeding
TORCH	Rubella virus, cytomegalovirus and herpes simplex virus type I IgG positive.	Negative	Excludes TORCH infection
Blood culture	Negative	Negative	
Cranial ultrasound	Normal	Normal	Indicates no evidence of intracranial hemorrhage
Echocardiogram	open foramen ovale (*φ* 1 mm)	Normal	

TORCH: “T” stands for Toxoplasmosis, “O” for Other infections (such as Herpes simplex virus type I and herpes simplex virus type II.), “R” for Rubella, “C” for Cytomegalovirus (CMV).

## Discussion

Intestinal malrotation with midgut volvulus represents a true surgical emergency in neonates, classically presenting with bilious vomiting and scaphoid abdomen within the first month of life (9, 10). Our case challenges this paradigm by demonstrating that hematochezia and hematemesis may serve as the earliest clinical manifestations, appearing within the critical first 48 hours after birth. This exceptionally rare presentation, not previously documented in the literature, carries significant implications for neonatal care.

The pathophysiology of early gastrointestinal bleeding in volvulus without necrosis warrants careful consideration. We propose a two-phase mechanistic model: initial venous congestion due to mesenteric torsion leads to mucosal injury and hemorrhage, while preserved arterial flow prevents transmural necrosis (4, 11). This hypothesis is supported by the patient's metabolic profile—elevated lactate (4.6 mmol/L) and negative base excess (−7.28) indicated tissue hypoxia, yet prompt intervention allowed complete recovery without bowel resection. Similar findings have been reported in fetal volvulus cases (4), suggesting a potential “pre-ischemic window” for intervention when bleeding precedes necrosis.

While upper gastrointestinal contrast remains the gold standard for diagnosing malrotation without volvulus (12), our experience underscores that point-of-care ultrasound—particularly the identification of the whirlpool sign—should be the first-line diagnostic tool when volvulus is suspected. This approach aligns with recent studies reporting 92%–100% sensitivity volvulus detection when performed by experienced operators (7, 13). Although ultrasound requires specialized training, its immediate availability at the bedside facilitates rapid diagnosis, while contrast studies—though more comprehensive for elective malrotation evaluation—often delay critical intervention in emergency settings due to their need for specialized equipment and radiology facilities. The absence of classic symptoms like scaphoid abdomen or bilious vomiting in our case aligns with reports that 1.79% of neonatal malrotation presents with isolated hematemesis and 12.5% presented hematochezia (9), underscores the limitations of conventional clinical criteria and emphasizes the need for a standardized diagnostic approach. Based on our findings and existing evidence (13–15), we propose the following protocol for neonates presenting with hematochezia: immediate bedside ultrasound by trained personnel should be performed as the first-line investigation (13), as it can reduce time to diagnosis by 68% (14). Neonatologists and pediatric surgeons should be proficient in emergency neonatal ultrasound. Training from experienced sonographers can help them conduct urgent scans when radiologists are unavailable, ensuring timely diagnosis and intervention to prevent bowel damage and save lives. Notably, 15% of volvulus cases lack abdominal signs, compelling emergent surgical assessment independent of exam findings (15). The management of intestinal malrotation requires urgent surgical consultation due to the significant risk of intestinal ischemia. The Ladd procedure remains the gold standard surgical approach, involving key steps of volvulus reduction, Ladd's band division, mesenteric base widening, and prophylactic appendectomy (15–19). Laparoscopic Ladd's procedure has gained popularity for its minimally invasive nature and faster recovery, but it faces challenges in neonates due to their small abdominal cavity and delicate tissues, potentially leading to higher conversion rates and complications (20–25). Despite some studies showing laparoscopic procedures to be superior in terms of operative time, hospital stay, and postoperative outcomes (26–28), the overall evidence remains inconclusive, with many surgeons still preferring the open approach due to its reliability and lower conversion rates (9, 29–32). In this case, we opted for an open Ladd's procedure due to the neonate's critical condition, which required immediate intervention. The severe midgut volvulus with a 480° twist necessitated precise surgical correction and assessment of bowel viability, which open surgery provides more reliably. Additionally, considering the limited intra-abdominal space in neonates and the potential for rapid deterioration, the open approach ensured optimal visualization and control, minimizing the risk of complications. This choice aligned with the surgical team's expertise and prioritized the safety and efficacy of the procedure, ultimately facilitating the patient's full recovery. Additionally, intraoperative fluid resuscitation is essential after volvulus reduction to counteract third-space losses and systemic inflammation triggered by reperfusion. Hemodynamic-guided crystalloid infusion helps maintain perfusion while avoiding volume overload. Close postoperative monitoring for metabolic acidosis and electrolyte imbalances is critical, as reperfusion injury can exacerbate tissue hypoxia through oxidative stress and microvascular dysfunction (33).

The application of artificial intelligence (AI) in ultrasound diagnosis shows promise for detecting intestinal malrotation, particularly where access to experienced radiologists is limited. While current evidence remains preliminary, AI may help identify key features like the whirlpool sign—Shakir et al. (34) demonstrated AI's ability to analyze complex ultrasound patterns, and Elyan et al. (35) highlighted its potential to recognize subtle imaging markers. Gumbs et al.'s Surgomics framework further supports integrating AI with clinical data for diagnostics (36). Future studies should validate AI specifically for whirlpool sign detection and malrotation diagnosis.

## Conclusions

Early-onset hematemesis and hematochezia in newborns must be taken seriously, even in the absence of other clinical signs, as these may represent early manifestations of intestinal malrotation. Prompt ultrasound diagnosis and immediate intervention can potentially salvage ischemic bowel segments and prevent the progression to intestinal necrosis.

## Data Availability

The original contributions presented in the study are included in the article/Supplementary Material, further inquiries can be directed to the corresponding author.
